# O-arm-guided percutaneous microwave ablation and cementoplasty for the treatment of pelvic acetabulum bone metastasis

**DOI:** 10.3389/fsurg.2022.929044

**Published:** 2022-09-12

**Authors:** Dongqing Zuo, Mengxiong Sun, Haoran Mu, Jiakang Shen, Chongren Wang, Wei Sun, Zhengdong Cai

**Affiliations:** ^1^ Department of Orthopedic Oncology, Shanghai General Hospital, Shanghai Jiao Tong University School of Medicine, Shanghai, China; ^2^ Shanghai Bone Tumor Institute, Shanghai, China

**Keywords:** pelvic bone metastasis, O-arm, microwave ablation, cementoplasty

## Abstract

**Objective:**

This study aims to evaluate the indications, safety, and efficacy of microwave ablation combined with cementoplasty under O-arm navigation for the treatment of painful pelvic bone metastasis.

**Methods:**

We retrospectively collected data from 25 patients with acetabulum bone metastasis who underwent microwave ablation combined with cementoplasty. All patients underwent percutaneous microwave ablation combined with cementoplasty under O-arm navigation. The postoperative follow-up included evaluations of pain, quality of life, function, the incidence of bone cement leakage, and the presence of perioperative complications. Pain and quality of life were evaluated using the visual analog scale (VAS) and the QLQ-BM22 quality of life questionnaire for patients with bone metastases, respectively. The functional scores were calculated using the MSTS93 scoring system of the Bone and Soft Tissue Oncology Society.

**Results:**

There were 10 males and 15 females with an average age of 52.5 ± 6.5 years, all 25 patients received percutaneous procedures, and no technical failure occurred. Major complications, including pulmonary embolism, vascular or nervous injury, hip joint cement leakage, and infection, were not observed in the current study. Pain regression was achieved in 24 of 25 patients. The mean VAS scores significantly decreased to 3.4 ± 1.0, 2.5 ± 1.2, and 1.2 ± 0.6 points at 1 week, 1 month, and 3 months after the procedure, respectively, compared with 7.0 points before the procedure (*P* < .05). The mean QLQ-BM22 score significantly decreased to 36.2 ± 4.9, 30 ± 5.6, and 25.4 ± 2.3 points at 1 week, 1 month, and 3 months after the procedure, respectively, compared with 55.8 points before the procedure (*P* < .05). The preoperative Musculoskeletal tumour society (MSTS) functional score of 25 patients was 18.5 ± 5.3 points, and MSTS score was 20.0 ± 3.0, 21.4 ± 4.9, and 22.8 ± 2.3 at 1 week, 1 month, and 3 months after the procedure, respectively (*P* < .05). The average bone cement injection volume was 8.8 ± 4.6 ml.

**Conclusion:**

The use of O-arm-guided percutaneous microwave ablation combined with cementoplasty for the treatment of pelvic metastases could quickly and significantly alleviate local pain, prevent pathological fracture, and improve the quality of life of patients with reduced complications.

## Introduction

The pelvis is one of the common sites of bone metastasis, which occurs in 20%–80% of advanced cancer patients (1, 2). The lung, breast, and prostate are the most common organs affected (3). Bone metastasis could result in skeletal-related events (SREs), including pain, pathological fracture, hypercalcemia, and nerve and visceral compression (4), which seriously affect the quality of life (QoL) of cancer patients, while more than 50% of patients received inadequate pain control treatment (5). With the development and advancement of various new anticancer drugs, next-generation sequencing, targeting, and immunotherapy, the treatment mode of patients with bone metastasis has gradually shifted to a chronic disease management mode. Long-term systemic follow-up and the continuous adjustment of drugs according to the patient's condition can confer long-term survival benefits, which is particularly important for improving tumor-bearing survival and QoL (6).

For pelvic bone metastases, surgical resection leads to the stripping of pelvic soft tissue and muscle attachment and addressing bone defects, causing more intraoperative bleeding, prolonging the healing time, and increasing the risk of wound complications, which in turn result in a longer recovery period for patients who often have a limited life expectancy (7). In some cases, in which medium-sized pelvic metastases, poor control after radiotherapy, local pain, and pathological fracture occur, surgical intervention can yield new problems, such as excessive bleeding, poor physical tolerance, the interruption of systemic treatment and radiotherapy, and adverse effects that result in the poor control of systemic tumors. Therefore, less invasive procedures, including percutaneous microwave ablation, radiofrequency ablation, cryoablation, and high-energy ultrasound with or without bone cement injection, have become salient options (8). While microwave ablation technology has been used for the treatment of bone tumors for more than 30 years and could be employed as an independent percutaneous minimally invasive treatment for benign bone tumors like Osteoid osteoma (9) and bone metastases (10). It has also been used as an intraoperative adjuvant treatment for the emergent control of intraoperative life-threatening tumor hemorrhage (11). In addition, tumor ablation can help to improve the safety margins during tumor resection (12). Yu et al. (13) took the lead in organizing and issuing the clinical guidelines for microwave ablation of bone tumors in limbs, which provided a good theoretical reference for microwave ablation of bone tumors and made the application of microwave ablation in bone tumors in limbs more standardized and professional. Percutaneous cementoplasty for the acetabulum was introduced by researchers (14–16). In addition, in the previous literature, pain control effects have been consistently reported (17–20), with common complications such as cement leakage and cement embolism. However, there has been no consensus and guidelines regarding how to reduce complications in the palliative treatment of pelvic bone metastasis, especially in regards to cementoplasty and microwave ablation, with few relevant clinical reports available.

Our team has focused on minimally invasive treatment of bone metastases for decades (21). The purpose of the current study was to evaluate cases of O-arm-guided microwave ablation combined with cementoplasty for the treatment of acetabular bone metastasis performed at our center in recent years to summarize the relevant indications, surgical methods, safety, and postoperative efficacy, with the aim of popularizing these technologies and sharing our clinical experience.

## Materials and methods

The indications were as follows: advanced cancer patients, (1) who had refractory pain caused by pelvic bone metastasis, did not respond to conservative treatment, (2) advanced cancer patients with an osteolytic lesion (in predominance) in the weight-bearing area around acetabulum suitable for the pain, (3) patients with limited life expectancy (less than 3–5 years). There was no absolute contraindication of the procedure; however, the relative contraindications were as follows: (1) patients in poor condition who had an expected survival period of less than 3 months; (2) bone destruction of the internal iliac plate with soft tissue mass contaminating important organs, nerves and blood vessels.

A total of 25 cases of pelvic bone metastasis treated in our center from June 2018 to June 2020 were retrospectively analyzed. The average age of the patients was 52.5 ± 6.5 years, including 10 males and 15 females. There were six cases of primary lung cancer, four cases of breast cancer, two cases of colon cancer, three cases of renal cell carcinoma, three cases of liver cancer, two cases of gastric cancer, one case of thyroid cancer, two cases of prostate cancer, one case of myeloma, and one case of cervical cancer (Table 1). All patients received PET-CT, local x-ray, enhanced CT, and enhanced MRI before operation. The pathology of all patients was determined by postoperative pathology. According to the location of the focus, there were 14 cases of periacetabulum (P), five cases of Pubic ischium + periacetabulum (PI + P), and six cases of area illium + periacetabulum (I + P). There were 15 cases of simple osteolytic bone destruction and 10 cases of mixed bone destruction. There were 10 cases of oligiometastasis and 15 cases of multiple metastases. All patients included in this series were evaluated by a multidisciplinary team from a quaternary care hospital, including specialists in oncology and radiation oncology, orthopedic surgery, and musculoskeletal interventional radiology. The majority of our patients were in palliative care after the failure of other treatments. All had received radiation therapy before the cementoplasty procedure and presented with persisting pain. This study was approved by the institutional ethics committee.

**Table 1:** Clinical characteristic of 25 patients.

Primary tumor	Number	Pelvic Enecking region	Pathology
Lung cancer	6	II (4), II + III (2)	Adenocarcinoma (2), neuroendocrine type (1), squamous cell carcinoma (2), adenosquamous cell carcinoma (1)
Prostate cancer	2	II (2)	Prostate adenocarcinoma 2
Breast cancer	4	II (2), II + III (2)	Intraductal carcinoma (2), invasive carcinoma (2)
Renal cancer	3	II (3)	Clear cell renal cell carcinoma (3)
Liver carcinoma	3	I + II (3)	Hepatocellular carcinoma 2, cholangiocarcinoma (1)
Gastric cancer	2	I + II (2)	Adenocarcinoma 1, squamous cell carcinoma (1)
Thyroid carcinoma	1	II (1)	Myeloid carcinoma (1)
Myeloma	1	II (1)	Myeloma (1)
Colorectal cancer	2	II + III (2)	Adenocarcinoma 1, undifferentiated carcinoma (1)
Cervical carcinoma	1	II (1)	Squamous cell carcinoma (1)

**Figure 1:**
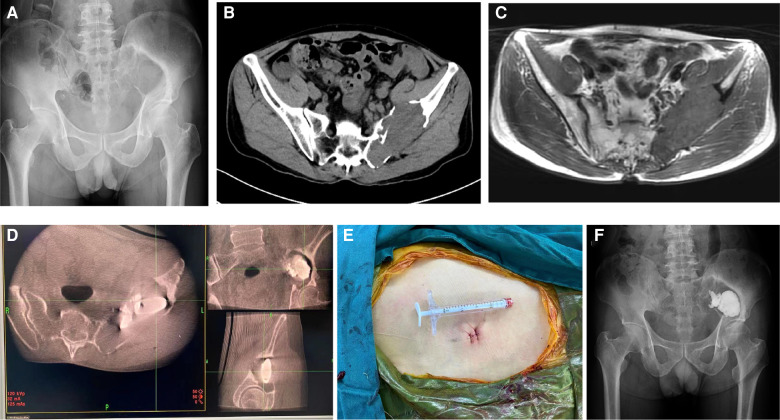
55-year-old man with metastatic renal cell carcinoma and a painful pelvic metastasis. (**A**) Preoperative pelvic x-ray. (**B,C**) Preoperative pelvic CT and MRI scans. (**D**) Intraoperative O-arm 3D image during puncture and cementoplasty. (**E**) Skin incision. (**F**) Postoperative pelvic x-ray 6 months after the procedure.

**Figure 2:**
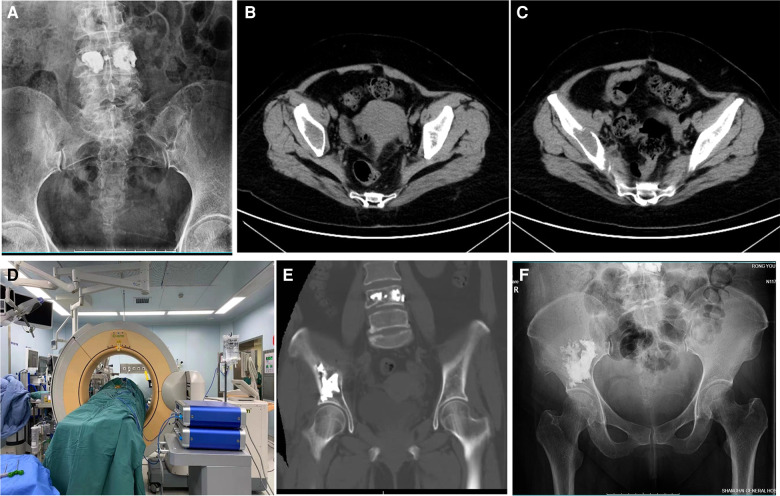
59-year-old man with metastatic liver carcinoma and a painful pelvic metastasis. (**A**) Preoperative pelvic x-ray. (**B,C**) Preoperative pelvic CT scan. (**D**) Intraoperative O-arm 3D image during puncture and cementoplasty. (**E**) Postoperative pelvic CT scan. (**F**) Postoperative pelvic x-ray 6 months after the procedure.

All patients underwent routine preoperative skin and bowel clearing preparation. General anesthesia with endotracheal intubation was applied in the supine position on a carbon spine bed (Allen, Hill Rock Company, MA, USA). For I + P bone metastasis, a lateral iliac plate approach approximately 2 cm above the acetabular roof is usually employed for puncture to avoid femoral nerve and vessel injury. Trocar needle positioning was then confirmed under O-arm navigation using a 3D mode scan. After the tumor tissue biopsy, the microwave needle (Nanjing Viking Jiuzhou) was inserted, and the radiographic reconfirmed. The microwave frequency was set to 2.45 kmHz. The insertion depth and position of the microwave needle were set according to the preoperative CT scan. After ablation applicator positioning through the trocar, the ablation procedure was conducted according to the protocol supplied by the equipment manufacturer. Single intraosseous lesions are routinely ablated with 60 W for 5–10 min; if the tumor diameter is larger than 5 cm, the position of the microwave needle can be adjusted for repeated ablation. For mixed lesion bone lesion, repeated ablations with multiple needles are recommended, usually, the bone cement injection should be very careful because of the high pressure intraosseous and high risk of cement leakage. During the ablation process, the ablation applicator is used with ice-cold saline-saturated gauze at the puncture site to protect the surrounding soft tissue. After microwave thermal ablation, the biopsy was confirmed to check the thermal ablation efficacy. Pelvic cementoplasty is used for pain management and bone reinforcement in certain cases of pelvic bone fractures and metastasis. Polymethyl methacrylate (PMMA) was then mixed and injected under O-arm real-time imaging control through the trocar, and the injection was suspended when satisfactory filling was obtained or leakage was detected (Figures 1 and 2).

According to the size of the bone metastasis, the patients were given antibiotics and methylprednisolone therapy intravenously 1–2 times after the operation, and pain relief and 24-h continuous hydration and alkalization treatment were also employed to protect renal damage after the absorption of ablation-induced necrosis. All patients were followed up before the operation, as well as 1 week, 1 month, and 3 months after the operation. The VAS pain score, QLQ-BM22 quality of life score (22), MSTS93 function score, and imaging evaluation were performed.

### Statistical analysis

The clinical parameter is expressed as the mean ± SD. Statistical analysis was carried out by GraphPad Prism software. The VAS and functional outcomes comparison was performed using the Tukey's multiple comparisons test. *P* < .05 indicated that the difference was statistically significant.

## Results

All 25 patients received percutaneous procedures with no technical failure or major complications. All procedures were completed by experienced doctors in our team. The average operation duration was 45 ± 18 min, the ablation power was 60 W, the average microwave ablation time was 8 min, and the average bone cement filling volume was 8.8 ± 4.6 ml. Pain regression was achieved in 24 of 25 patients and one patient experienced recurrent pain caused by pelvic bone metastasis and received a repeat procedure. Posttreatment radiographs did not reveal osteolysis in the area of cementation, bone cement dislocation, or loosening within the acetabular bone. Pathological fracture within the strengthened acetabulum was not found. No one was reverted to secondary open reconstruction surgery.

### Surgical-related complications

Major complications, including pulmonary embolism, vascular or nervous injury, hip joint cement leakage, and infection, were not observed in the current study. One patient with acetabular roof ablation encountered ablation needle fracture due to direction adjustment during the ablation process and received open surgery for foreign bodies removal. One patient had local wound problems one month after I + P ablation and underwent debridement. Six of 25 patients experienced transient hip pain during anesthesia-induced anabiosis (lasting 2 h) and received 100 mg of methylprednisolone, which is considered to elicit a thermal effect on the nerves behind the acetabulum, to relieve pain soon after treatment. There were no other complaints or discomfort after the operation.

### Postoperative pain and functional outcome

**Figure 3:**
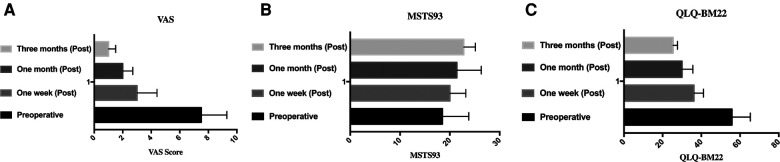
VAS, MSTS93, and QLQ-BM22 scores before and after operation. (**A**) VAS score in preoperative, 1 week, 1 month, and 3 months postoperative. (**B**) MSTS93 in preoperative, 1 week, 1 month, and 3 months postoperative. (**C**) QLQ-BM22 score in preoperative, 1 week, 1 month, and 3 months postoperative. VAS, visual analog scale.

The mean VAS scores significantly decreased to 3.4 ± 1.0, 2.5 ± 1.2, and 1.2 ± 0.6 points at 1 week, 1 month, and 3 months after the procedure, respectively, compared with 7.0 ± 1.8 points before the procedure (*P* < .05 for all pairs). While the mean QLQ-BM22 scores significantly decreased to 36.2 ± 4.9, 30 ± 5.6, and 25.4 ± 2.3 points at 1 week, 1 month, and 3 months after the procedure, respectively, compared with 55.8 ± 9.5 points before the procedure, the quality of life of patients was significantly improved, and the difference was statistically significant (*P* < .05 for all pairs). The preoperative MSTS score of 25 patients was 18.5 ± 5.3 points, and MSTS score was 20.0 ± 3.0, 21.4 ± 4.9, and 22.8 ± 2.3 at 1 week, 1 month, and 3 months after the procedure, respectively (*P *< .05 for all pairs) (Table 2, Figure 3).

**Table 2:** Pain, function and QoL score.

Parameter/Time	Preoperative	1 week (post)	1 month (post)	3 months (post)
VAS score	7.0 ± 1.8	3.4 ± 1.0	2.5 ± 1.2	1.2 ± 0.6
MSTS score	18.5 ± 5.3	20.0 ± 3.0	21.4 ± 4.9	22.8 ± 2.3
EORTC QLQ-BM22	55.8 ± 9.5	36.2 ± 4.9	30 ± 5.6	25.4 ± 2.3

VAS, visual analog scale.

## Discussion

It is difficult to monitor the adjacent critical structures of the pelvis during minimally invasive procedures of pelvic bone metastasis due to its complex anatomy, which requires a high level of experience and professional equipment. Medtronic's O-arm system is a new generation of intraoperative imaging platforms that are perfectly compatible with real-time three-dimensional (3D) images for surgery (23). To our knowledge, this is the first study using a strategy of O-arm-guided percutaneous microwave ablation and pelvic cementoplasty to treat painful pelvic bone metastasis, the results are in line with those described in the literatures. Through preoperative and intraoperative imaging measurements, we can implement accurate microwave ablation and cementoplasty to achieve satisfactory bone reinforcement, pain alleviation, greatly improved safety, and postoperative functional efficacy with the O-arm system. Kim et al. (8) found that local complications and extraosseous bone cement leakage were often observed (36%, 72/201 of pelvises). Among them, 21 showed intraarticular leakage into the hip joint, and 51 showed leakage into areas other than the hip joint. Moser et al. (19) reported that cement leakage was absent for 24 lesions (54.5%), articular leakage was absent for 6 lesions (13.6%), muscular or venous leakage was absent for 13 lesions (29.5%), and foraminal leakage was absent for one sacral lesion (2.3%). Compared with the procedures of previous studies carried out with CT device or mobile C-arm unit or in an angiography suite, the current study used O-arm-guided technology to monitor percutaneous ablation and cement bone reinforcement, and there were no major perioperative complications in this group. Only one patient had intraoperative leakage of the ablation needle. Six of 25 patients experienced transient hip pain, which was relieved soon after returning to the ward. And the procedure could be more efficient with its compatible navigation system, like the Medtronic stealthStation S8 system, the real-time image will not only greatly decrease the radiation exposure for patients and surgeons, but also help to reduce puncture-related risks. The combination procedure significantly reduced the operation time and local tumor contamination (compared with unpublished data), promoted rapid postoperative recovery, significantly relieved pain, and allowed most patients to receive systemic treatment of tumors in a short time with fewer complications compared with previous studies. The results of the current study suggested that O-arm-guided percutaneous microwave ablation and cementoplasty can effectively enhance the stability of the iliac and acetabulum in patients with pelvic bone metastases with little risk of complication.

Microwaves can be used to ablate tumors by agitating polar water molecules in the tumor tissue. The friction of water molecules produces heat of approximately 100–120 °C, thus inducing cellular death *via* coagulation necrosis. Compared with radiofrequency ablation, it has the advantage of rapid temperature rise, high temperature in the tumor, short time, little influence by carbonized blood flow, and no influence by impedance, so it has made great progress in its clinical application. Several studies have reported microwave ablation in bone tumor treatment, but there are only a few studies on the setting of ablation time and temperature parameters of microwave ablation in bone tumors (24, 25).

In this study, we adopted different ablation and bone cement strengthening strategies for pelvic metastatic lesions with different sizes and bone destruction forms: (1) For lesions with complete internal iliac plates, the lesions can be ablated repeatedly for 5 min, and bone cement strengthening can be performed after biopsy. (2) For patients with partial defects of the internal iliac plate, it is recommended to directly form bone cement after ablation for 3–5 min to reduce the risk of internal cement leakage and pelvic organ injury. (3) For patients with large lesions involving both internal and external plates, percutaneous ablation is not recommended. Local open surgery under the control of an abdominal aortic balloon or embolization is recommended. Furthermore, we usually do not endorse inserting two or more needles for ablation or cement filling, partly because of the low cost-effect ratio of microwave ablation in bone metastasis treatment, the other reason is multiple uses of ablation needles increase the risk of cement leakage and other complications.

The limitation of this study, however, is the retrospective design and the small cohort of patients may represent a limit to the statistical analysis, which is due to the specific location and scarcity nature included in our study, which also caused the lack of control cohort is current study, which could be solved by designing prospective comparative clinical trials for the combinations of those procedures. One of our limitations, however, is the compatibility between the biopsy kit and the microwave ablation trocar; there is currently no commercially available kit that is compatible for bone biopsy, microwave, and bone cement strengthening; we had to change the trocar to finish the procedure, which definitely added to the risk of bone destruction and cement leakage, but also increased the risk of re-puncture positioning. And we are now working on another project to develop a more compatible bone biopsy kit in order to overcome those limitations and push forward the palliative treatment.

In summary, we confirmed the safety and efficacy of the combination of percutaneous microwave ablation and cementoplasty under O-arm navigation in terms of pain relief and recovery of their QoL. Moreover, this strategy has a low risk of complications compared with radiofrequency ablation or cryoablation with or without an intraoperative CT scan.

## Data Availability

The original contributions presented in the study are included in the article/Supplementary Material, further inquiries can be directed to the corresponding author/s.
